# Gene expression analysis reveals early changes in several molecular pathways in cerebral malaria-susceptible mice versus cerebral malaria-resistant mice

**DOI:** 10.1186/1471-2164-8-452

**Published:** 2007-12-06

**Authors:** Nicolas F Delahaye, Nicolas Coltel, Denis Puthier, Mathieu Barbier, Philippe Benech, Florence Joly, Fuad A Iraqi, Georges E Grau, Catherine Nguyen, Pascal Rihet

**Affiliations:** 1 Laboratoire de Pharmacogénétique des maladies parasitaires-EA864, Université de la Méditerranée, IFR48, Marseille, France; 2 Université de la Méditerranée-IFR48, CNRS-UMR 6020-Immunopathology group, Marseille, France; 3 INSERM ERM 206-TAGC, Université de la Méditerranée, IFR137, Marseille, France; 4 Tel-Aviv University, Department of human microbiology, Sackler Faculty of Medicine, Ramat-Aviv, Tel-Aviv 69978, Israel; 5 University of Sydney, Department of Pathology, Faculty of Medicine and Bosch Institute, Australia; 6 Institut Gustave Roussy, Villejuif, France

## Abstract

**Background:**

Microarray analyses allow the identification and assessment of molecular signatures in whole tissues undergoing pathological processes. To better understand cerebral malaria pathogenesis, we investigated intra-cerebral gene-expression profiles in well-defined genetically cerebral malaria-resistant (CM-R) and CM-susceptible (CM-S) mice, upon infection by *Plasmodium berghei *ANKA (PbA). We investigated mouse transcriptional responses at early and late stages of infection by use of cDNA microarrays.

**Results:**

Through a rigorous statistical approach with multiple testing corrections, we showed that PbA significantly altered brain gene expression in CM-R (BALB/c), and in CM-S (CBA/J and C57BL/6) mice, and that 327 genes discriminated between early and late infection stages, between mouse strains, and between CM-R and CM-S mice. We further identified 104, 56, 84 genes with significant differential expression between CM-R and CM-S mice on days 2, 5, and 7 respectively. The analysis of their functional annotation indicates that genes involved in metabolic energy pathways, the inflammatory response, and the neuroprotection/neurotoxicity balance play a major role in cerebral malaria pathogenesis. In addition, our data suggest that cerebral malaria and Alzheimer's disease may share some common mechanisms of pathogenesis, as illustrated by the accumulation of β-amyloid proteins in brains of CM-S mice, but not of CM-R mice.

**Conclusion:**

Our microarray analysis highlighted marked changes in several molecular pathways in CM-S compared to CM-R mice, particularly at early stages of infection. This study revealed some promising areas for exploration that may both provide new insight into the knowledge of CM pathogenesis and the development of novel therapeutic strategies.

## Background

Malaria is a disease affecting millions of people worldwide. Cerebral malaria (CM) is one of the most severe complications and is a major cause of death. Both host and parasite genetic factors play important roles in the outcome of malaria infection. Epidemiological data, candidate gene studies, and genetic linkage studies clearly support the existence of a genetic contribution to susceptibility to human malaria [1]. In parallel with human studies, malaria susceptibility genes have been mapped in mouse models, and the role of some genes has been demonstrated [2]. It is clear, however, that a number of malaria susceptibility genes remain to be identified. These include genes, whose expression is likely deregulated upon malaria infection.

Transcriptional profiling may provide new tools for identifying the key genes that govern host responses against pathogens. Recently, several reports have described gene expression changes that accompany the host response against Plasmodium *spp. *Microarrays have been analyzed from mice [3-5], rhesus monkey [6] and humans [7], upon infection by Plasmodium *spp *A parallel can be observed in the regulation of genes involved in immune responses, glycolysis, and erythropoiesis. These data suggested that variation in host gene expression may be associated with resistance or susceptibility to malaria.

Recently, we investigated brain gene expression patterns in well-defined genetically CM-resistant (CM-R) and CM-susceptible mice (CM-S) by use of cDNA microarrray [8]. We identified a set of genes that perfectly discriminates between CM-R and CM-S mice at the time of CM onset. This indicates that gene expression analysis using microarray tools may be useful for the identification of candidate genes that are potentially responsible for resistance or susceptibility to CM. Nevertheless, an important issue was to identify genes whose expression differ between CM-R and CM-S mice before the time of CM onset to identify early events that may participate in malaria pathogenesis. In this report, we present an analysis of genes differentially expressed in brains from CM-R and CM-S mice prior to infection, and at the early and late stages of infection with *Plasmodium berghei *ANKA (PbA). Data analysis reveals that molecules belonging to several biological processes were preferentially and differentially expressed between CM-R and CM-S mice, and that a number of gene expression changes occurred at the early and late stages of infection. Herein, we discuss new working hypotheses on this basis.

## Results

### Identification of genes regulated in brains by PbA infection

The ANOVA of microarray data revealed significant gene expression changes over the course of infection in BALB/c (n = 25) mice, CBA/J mice (n = 16), and in C57BL/6 mice (n = 20). We calculated empirical *P *values for each gene, and we considered *P *< 0.05 significant. On this basis, we selected 174, 210, and 342 genes for BALB/c mice, CBA/J mice, and C57BL/6 mice, respectively.

**Figure 1:**
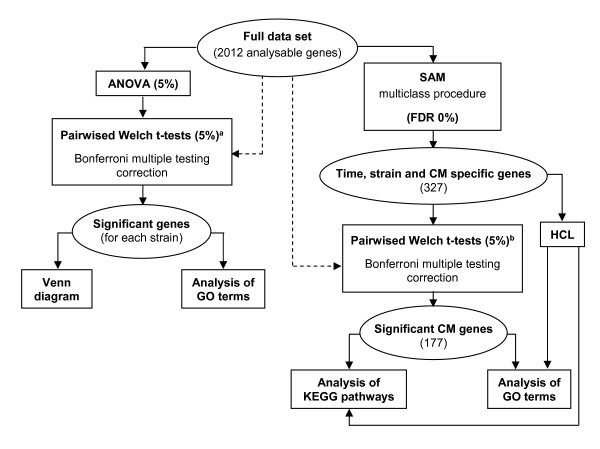
**Schematic outline of data analysis**. HCL: hierarchical clustering. ^a ^Brain gene expression prior to infection was compared with brain gene expression on days 2, 5 and 7. ^b ^Brain gene expression in CM-R mice was compared with that in CM-S mice at each time point. As represented by dashed arrows, we considered the whole data set (n = 2012 genes) to carry out multiple testing correction.

**Figure 2:**
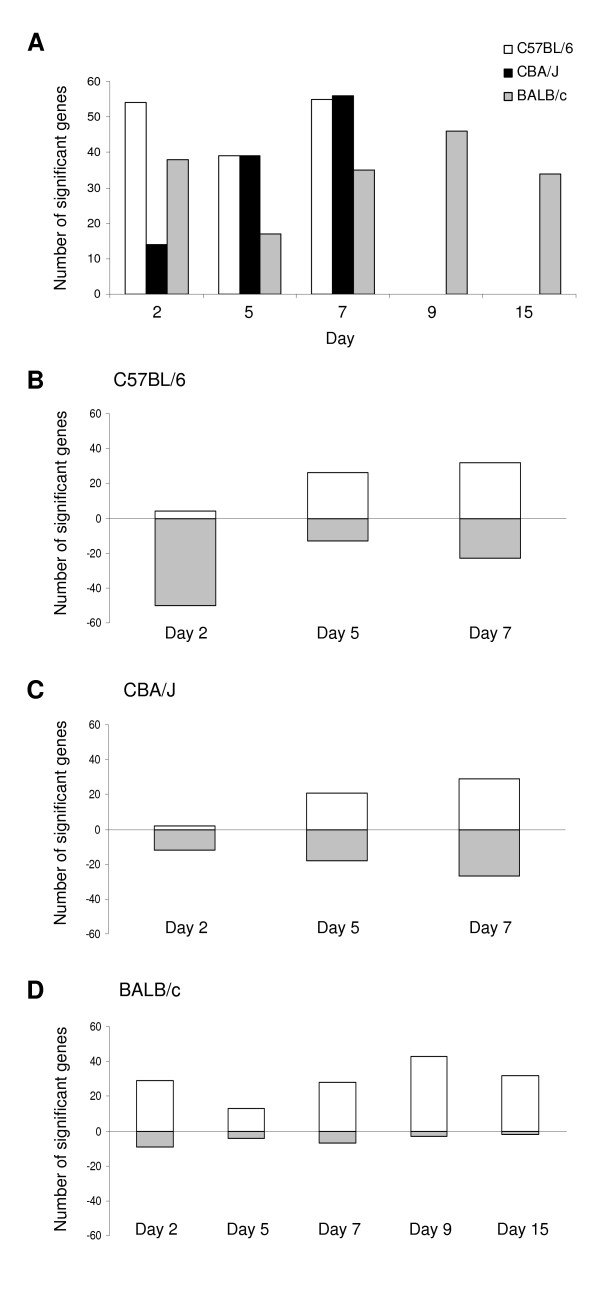
**Distribution of genes regulated by PbA infection according to the time of infection.** The genes whose expression was significantly altered by PbA infection were identified by using pair-wise Welch t tests with a Bonferroni correction.**A**. The number of genes with significant changes is shown at each time point for each mouse strain. **B**, **C **and **D**. The number of genes significantly up- (positive values) and down-regulated (negative values) is shown at each time point for each mouse strain.

To further compare uninfected mice with infected mice, we performed a Welch t test and we applied a Bonferroni correction to account for multiple tests performed (Figure 1). Figure 2 shows the number of genes whose expression was significantly altered by PbA infection. The mouse strains displayed various patterns. Strikingly, the number of genes that showed significant expression changes was higher in C57BL/6 mice than in CBA/J mice on day 2 post-infection with PbA (Figure 2A). The number of genes that showed significant expression changes in CBA/J mice gradually increased during infection. In contrast, the number of genes that showed significant expression changes in both C57BL/6 mice and BALB/c mice on day 2 was similar to the number of genes identified on day 7; it was, nevertheless, lower on day 5 than on days 2 and 7. As shown in Figure 2, most of the genes identified in C57BL/6 mice (Figure 2B) and in CBA/J mice (Figure 2C) were under-expressed, while most of the genes identified in BALB/c mice (Figure 2D) were over-expressed.

**Figure 3:**
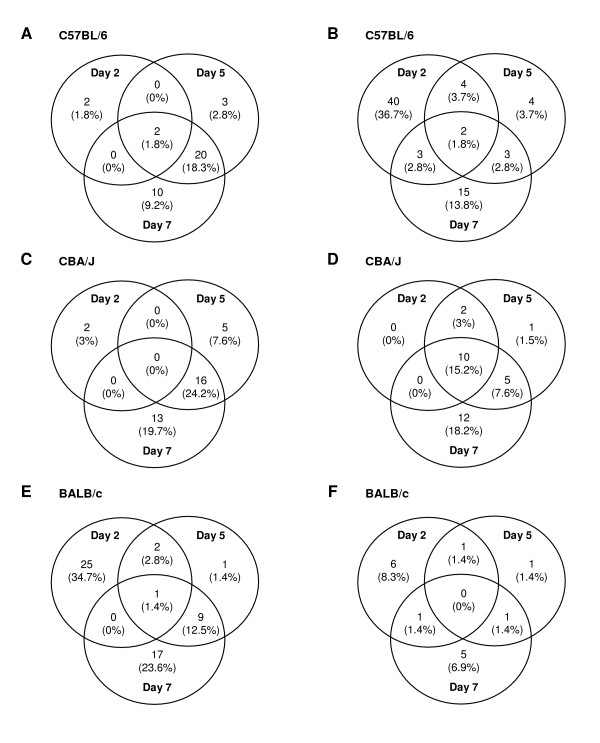
**Overlapping genes with significant differential expression at different time points during PbA infection.** This Venn diagrams show the number of overlapping genes with significant expression changes on days 2, 5, and 7 post-infection with PbA, for C57BL/6 mice (**A **and **B**), CBA/J mice (**C **and **D**) and BALB/c mice (**E **and **F**). Genes that are either induced or suppressed by infection are distinguished for each strain: the number of genes induced is shown in **A**, **C**, and **E**, while the number of genes suppressed is shown in **B**, **D**, and **F**.

A Venn diagram summarizes the number of overlapping genes with significant differential expression at different time points (Figure 3). Most of the genes identified on day 2 were no longer identified on day 7 in C57BL/6 mice and in BALB/c mice. As shown in Figure 3A–B and 3E–F, 42 of 53 and 31 of 36 genes with differential expression on day 2 did not show differential expression on days 5 and 7 in C57BL/6 and BALB/c mice, respectively. In contrast, 10 of 14 genes regulated by PbA infection on day 2 were also regulated on days 5 and 7 in CBA/J mice (Figure 3C–D), indicating that C57BL/6 mice and CBA/J mice partly differ in their transcriptional response over the course of infection.

### Microarray analysis discriminates between CM-R and CM-S mice according to the time after infection

We searched for genes showing differential expression between mouse strains before infection. The ANOVA identified 125 genes without multiple test correction (data not shown), and 7 genes with Bonferroni multiple test correction at the level of 5%: 1700023B02Rik, Acot8, Gpd2, Sec11c, Ngly1, Zfp346 and Tln2. These results revealed a minor natural variation in gene expression between mouse strains. Nevertheless, we took into account this natural variation in further analyses to search for transcriptional changes. Thus, the individual gene expression level in infected mice was adjusted for the gene expression level in uninfected mice for each mouse strain.

**Figure 4:**
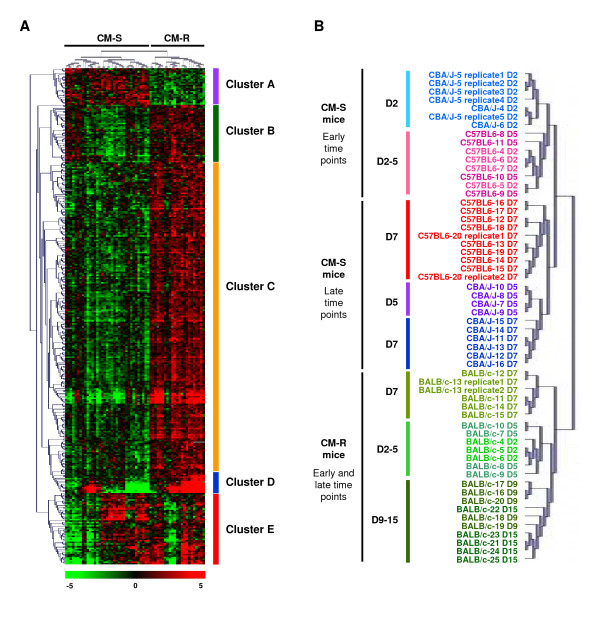
**Hierarchical classification of mouse-strain-specific and CM-R/CM-S genes according to the time of infection**. **A**. Hierarchical clustering of the 58 brain tissue samples, using expression levels of 327 significant genes differentially expressed between the mouse strains at early and late stages of infection. This set of genes was extracted from the full data set (n = 2012) by use of a SAM procedure and a false discovery rate of 0%. Each row represents a gene and each column represents a sample. Red and green indicate expression levels above and below the median, respectively. Grey indicates missing data. Dendograms of samples (above matrix) and genes (to the left of matrix) represent overall similarities in gene expression profiles. **B**. Dendogram of samples representing the results of the same global hierarchical clustering applied to the 58 brain tissue samples. Clustering of technical replicates (CBA/J-5, C57BL6-20 and BALB/c-13) is shown: the samples taken from a CBA/J mouse on day 2, a C57L/6 mouse on day 7, and a BALB/c mouse on day 7 were run on 5, 2, and 2 microarrays, respectively. Samples taken from mice on days 2, 5, 7, 9, and 15 post-infection are coded D2, D5, D7, D9, and D15, respectively.

To focus on transcriptional changes associated with resistance or susceptibility, we searched for a set of genes that discriminated between CM-R mice (BALB/c) and CM-S mice (C57BL/6 and CBA/J). To this end, we used the multi-class Significant Analysis of Microarrays (SAM) procedure, and we applied a false discovery rate of 0% (Figure 1). The analysis yielded a set of 327 genes [see Additional file 1], which was used to perform unsupervised hierarchical clustering (Figure 4A). Interestingly, the expression of several genes was induced in CM-R mice or suppressed in CM-S mice as a result of infection, while the expression of other genes was suppressed in CM-R mice and induced in CM-S mice [see Additional file 1]. This led to successfully classify CM-R and CM-S mice according to the time after infection (Figure 4B) and to determine five clusters (Figure 4A). Clusters A and C fully grouped CM-R mice (BALB/c) versus CM-S mice (C57BL/6 and CBA/J) whatever the time after infection. Genes of cluster A were over-expressed in CM-S mice compared to CM-R mice, while genes of cluster C were under-expressed in CM-S mice compared to CM-R mice. Clusters B and D also grouped CM-R mice versus CM-S mice. Nevertheless, clusters B and D discriminated between C57BL/6 and CBA/J mice, indicating differential gene expression between the two CM-S strains at early time points of infection. Genes of clusters B and D were over-expressed in CBA/J mice on day 2 post-infection compared to C57BL/6 mice, and in C57BL/6 on days 2 and 5 post-infection compared to CBA/J mice, respectively. At late stage of infection, genes of clusters B and D showed similar gene expression between the two CM-S strains, and were under-expressed in CM-S mice compared to CM-R mice. Cluster E discriminated mice at early and late stage of infection. Nevertheless, genes of cluster E were over-expressed in CM-S mice compared to CM-R mice indicating an interval between the response of CM-S and the response of CM-R mice.

**Figure 5:**
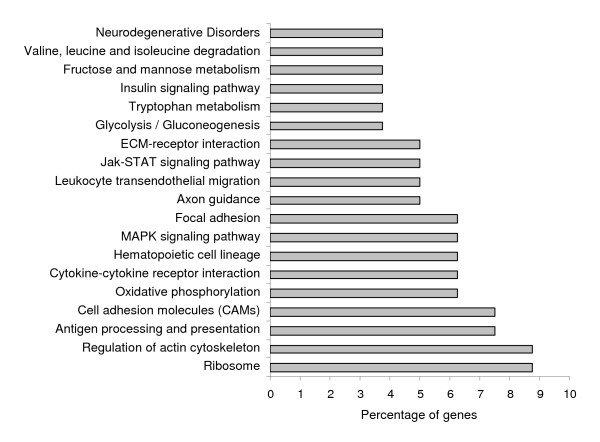
**View of biological functional annotation repartition of the genes grouped in clusters B and C**. The KEGG pathways, in which the genes were known to be involved, are shown. Of the 245 genes of the clusters B and C, 80 were annotated. We represented only the KEGG pathways that contained at least three genes.

To analyze functional annotations related to CM, we sought biological process Gene Ontology (GO) terms and KEGG pathways for the 327 genes that discriminated between CM-R mice (BALB/c) and CM-S mice (C57BL/6 and CBA/J). The analysis of biological process GO terms of gene clusters showed an over-representation of some GO terms. In particular, the GO terms related to the "defense response" category, such as the "immune response" or the "inflammatory response" GO terms were strongly over-represented in cluster E (Table 1). Similarly, most of the genes grouped in cluster E were found to be involved in KEGG pathways related to immune responses, such as "cytokine-cytokine receptor interaction" or "natural killer cell mediated cytotoxicity". The analysis of other clusters did not reveal, however, a strong over-representation of particular GO terms, suggesting that these clusters were heterogeneous. In particular, clusters B and C contained 38 and 207 genes with heterogeneous GO categories. This was further supported by the analysis of KEGG pathways, which pointed out a number of different pathways, such as metabolic energy pathways ("glycolysis/gluconeogenesis" and "oxidative phosphorylation"), immune responses ("cytokine-cytokine receptor interaction" and "antigen processing and presentation"), haematopoiesis, ("hematopoietic cell lineage"), cytoskeleton pathways ("regulation of active cytoskeleton"), or pathways related to brain function ("neurodegenerative disorders" and "axon guidance") (Figure 5).

**Table 1:** Enrichment of the 327 genes differentially expressed^a ^in Gene Ontology terms

Cluster, GO term	Fisher exact test^b^
Cluster A	
Defense response^c^	0.043
Cluster B	
...	...
Cluster C	
Positive regulation of cell activation^c^	0.0024
Positive regulation of lymphocyte	0.0024
activation^c^	
Positive regulation of cellular	0.006
physiological process^c^	
Regulation of phosphorylation^c^	0.008
Cell death	0.013
Positive regulation of immune response	0.015
Positive regulation of cellular	0.036
biosynthesis	
Cytokines and inflammatory response	0.049
Cluster D	
...	...
Cluster E	
Defense response	0.0000001 ^d^
Immune response	0.000004^d^
Response to pest/pathogen/parasite	0.0000051^d^
Inflammatory response	0.0027
Response to stress	0.0028
Response to wounding	0.003
Chemotaxis	0.0054
Response to external stimulus	0.011

^a ^This set of genes was extracted from the full data set (n = 2012) by use of a SAM procedure and a false discovery rate of 0%.

^b ^Significant results with a Fischer exact test (P < 0.05).

^c ^GO terms that were also over-represented in the set of 177 significant CM genes.

^d ^Significant results with the Benjamini multi-testing correction (P < 0.05).

**Figure 6:**
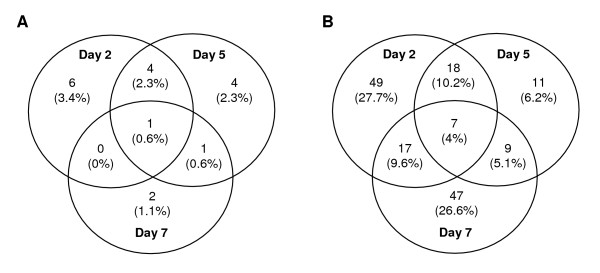
**Genes differentially expressed between CM-R and CM-S mice on days 2, 5, and 7.** The number of genes up-regulated in CM-S mice compared to CM-R mice (**A**), and the number of genes up-regulated in CM-R mice compared to CM-S mice (**B**) are shown.

We specifically searched for genes with significant differential expression between CM-R and CM-S mice. Thus, we used the Welch t test to compare BALB/c mice (CM-R mice) on the one hand and CBA/J mice and C57BL/6 mice (CM-S mice) on the other hand (Figure 1). We tested the 327 genes identified by the SAM multi-class procedure, but we considered the whole data set (n = 2012 genes) to carry out multiple testing correction. Among the 327 genes, 177 genes were further found to be differentially expressed between CM-R mice and CM-S mice (Table 2). Among the 177 genes, 104, 56, and 84 genes showed significant differential expression on days 2, 5, and 7, respectively, and 11, 17, 138, and 11 were grouped in clusters A, B, C and E, respectively. The number of co-occurring genes with differential expression between CM-R and CM-S mice detected at different times is displayed in Figure 6. Eighteen of 177 genes were up-regulated in CM-S mice compared to CM-R mice, while 158 of 177 genes were up-regulated in CM-R mice compared to CM-S mice. Only one gene showed a complicated picture: Fyco1 was down-regulated in CBA/J and C57BL/6 mice (CM-S mice) mice on day 2, and in CBA/J mice on day 5, while it was up-regulated in C57BL/6 mice on day 5. Table 3 show the most represented KEGG pathways, which included pathways related to metabolism, erythropoiesis, immune responses, neuronal development, and neurodegenerative disorders.

**Table 2:** *C*M-specific genes obtained by use of the Welch t test and a Bonferroni correction

ID^a^	Cluster	Symbol^b^	Name	Chr^c^	Welch t test^d^		
BG083931	A	Slc25a11	Solute carrier family 25 (mitochondrial carrier; oxoglutarate carrier), member 11	11	D2	...	...
718084	A	Dfy	Duffy blood group	1	D2	...	...
BG064714	A	Slc3a2	Solute carrier family 3 (activators of dibasic and neutral amino acid transport), member 2	19	D2	...	...
573549	A	Mrps34	Mitochondrial ribosomal protein S34	17	D2	D5	...
641184	A	Cdc37l1	Cell division cycle 37 homolog (S. cerevisiae)-like	19	D2	D5^e^	...
641058	A	Nfkbia	Nuclear factor of kappa light chain gene enhancer in B-cells inhibitor, alpha	12	...	D5	D7
BG086014	A	Hmgn1	High mobility group nucleosomal binding domain 1	16	D2	D5	...
639606	A	E030041M21Rik	RIKEN cDNA E030041M21 gene	10	D2^f^	D5	...
636468	A	Epb4.1l3	Erythrocyte protein band 4.1-like 3	17	...	D5	...
BG084377	A	Stat3	Signal transducer and activator of transcription 3	11	D2	...	...
582660	A	Tagap1	T-cell activation GTPase activating protein 1	17	D2	D5	D7
573243	B	Ddi2	DNA-damage inducible protein 2	4	...	D5	...
BG071164	B	2310008M10Rik	RIKEN cDNA 2310008M10 gene	3	D2	...	D7
BG070859	B	Nipbl	Nipped-B homolog (Drosophila)	15	...	D5^e^	D7
639002	B	Mlx	MAX-like protein X	11	D2	D5	D7
BG072955	B	Myo5a	Myosin Va	9	...	...	D7
575433	B	Ptger1	Prostaglandin E receptor 1 (subtype EP1), 42 kD	8	...	...	D7
BG087138	B	Itsn2	Intersectin 2	12	D2	...	D7
BG078795	B	Hspa5	Heat shock 70 kD protein 5 (glucose-regulated protein)	2	...	...	D7
BG064608	B	Calr	Calreticulin	8	...	...	D7
BG087169	B	P4hb	Protein disulfide isomerase associated 6	12	...	...	D7
764825	B	Reln	Reelin	5	D2	...	...
1263789	B	Cxcl12	Chemokine (C-X-C motif) ligand 12	6	D2	...	...
441212	B	Atp6v1g2	ATPase, H+ transporting, lysosomal 13 kD, V1 subunit G isoform 2	17	D2	...	...
577991	B	Zdhhc6	Zinc finger, DHHC domain containing 6	19	...	D5	...
1263101	B	Hmgcs1	3-hydroxy-3-methylglutaryl-Coenzyme A synthase 1	13	...	D5	...
573257	B	Pcgf3	Polycomb group ring finger 3	5	...	...	D7
436894	B	Fabp7	Fatty acid binding protein 7, brain	10	...	...	D7
BG076809	C	Rps18	Ribosomal protein S18	17	D2	...	...
BG085025	C	Dab1	Disabled homolog 1 (Drosophila)	4	...	D5	...
718873	C	S100a10	S100 calcium binding protein A10 (calpactin)	3	D2	D5	...
576647	C	Chchd1	Coiled-coil-helix-coiled-coil-helix domain containing 1	14	D2	D5	...
BG063799	C	Ak3l1	Adenylate kinase 3 alpha-like 1	4	...	...	D7
BG077012	C	Cdc6	Cell division cycle 6 homolog (S. cerevisiae)	11	D2	D5	...
BG077054	C	Ncbp2	Nuclear cap binding protein subunit 2, 20 kDa	16	D2	...	...
BG062969	C	Hnrpdl	Heterogeneous nuclear ribonucleoprotein D-like	5	D2	...	...
576635	C	Vcam1	Vascular cell adhesion molecule 1	3	D2	...	...
402711	C	Crybb1	Crystallin, beta B1	5	...	...	D7
BG080965	C	Prkcbp1	Protein kinase C binding protein 1	2	...	...	D7
BG074422	C	Itgb1	Integrin beta 1 (fibronectin receptor beta)	8	...	D5	...
BG084605	C	Bpgm	2,3-bisphosphoglycerate mutase	6	D2	...	...
BG078316	C	Sfxn1	Sideroflexin 1	13	...	...	D7
BG076061	C	Lyar	Ly1 antibody reactive clone	5	...	...	D7
BG077320	C	Mrps10	Mitochondrial ribosomal protein S10	17	D2	...	...
BG077670	C	Gnai2	Guanine nucleotide binding protein, alpha inhibiting 2	9	D2	...	D7
406838	C	Foxo3a	Forkhead box O3a	10	...	...	D7
BG072299	C	Ramp2	Receptor (calcitonin) activity modifying protein 2	19	...	...	D7
1224916	C	Atxn7	Ataxin 7	14	D2	...	...
1395654	C	Batf	Basic leucine zipper transcription factor, ATF-like	12	D2	D5	...
BG087349	C	Aldh1b1	Aldehyde dehydrogenase 1 family, member B1	4	D2	...	...
596637	C	Stab2	Stabilin 2	10	D2	...	...
596064	C	Cd5	CD5 antigen	19	D2	...	...
573880	C	Nfyc	Nuclear transcription factor-Y gamma	4	D2	...	...
1226020	C	Unknown	Unknown	...	D2	...	D7
400157	C	Uqcrq	Ubiquinol-cytochrome c reductase binding protein	11	...	...	D7
BG086406	C	Agtr2	Angiotensin II receptor, type 2	X	...	...	D7
582804	C	Cys1	Cystin 1	12	...	D5	...
576480	C	Igf2bp3	Insulin-like growth factor 2 mRNA binding protein 3	6	D2	D5	...
464586	C	Srr	Serine racemase	11	D2	...	...
575610	C	2700085E05Rik	RIKEN cDNA 2700085E05 gene	11	D2	...	...
575040	C	Sel1h	Sel1 (suppressor of lin-12) 1 homolog (C. elegans)	12	D2	...	...
BG087165	C	Herc4	Hect domain and RLD 4	10	D2	...	D7
BG073254	C	Rbmxrt	RNA binding motif protein, X chromosome retrogene	8	D2	...	...
BG073981	C	Gtpbp8	GTP-binding protein 8 (putative)	16	...	D5	D7
573285	C	Lrriq2	Leucine-rich repeats and IQ motif containing 2	16	D2	...	...
372621	C	Icam5	Intercellular adhesion molecule 5, telencephalin	9	...	...	D7
1428894	C	Ptpns1	Protein tyrosine phosphatase, non-receptor type substrate 1	2	...	...	D7
BG076772	C	Gpd2	Glycerol phosphate dehydrogenase 2, mitochondrial	2	...	...	D7
752149	C	Stk16	Serine/threonine kinase 16	1	...	D5	D7
573328	C	Thrsp	Thyroid hormone responsive SPOT14 homolog (Rattus)	7	...	D5	D7
BG087431	C	Sec31a	SEC31 homolog A (S. cerevisiae)	5	D2	...	...
BG087401	C	Brd7	Bromodomain-containing 7	8	D2	D5	D7
1361813	C	Mmachc	Methylmalonic aciduria cblC type, with homocystinuria	4	D2	...	...
BG076501	C	Sfrs6	Splicing factor, arginine/serine-rich 6	2	...	...	D7
1243669	C	Aldoa	Aldolase 1, A isoform	7	...	D5	...
476643	C	Sez6l	Seizure related 6 homolog like	5	D2	...	...
BG075757	C	Cd81	CD 81 antigen	7	D2	...	...
574496	C	Usp19	Ubiquitin specific peptidase 19	9	D2	D5^f^	...
BG065688	C	Uba52	Ubiquitin A-52 residue ribosomal protein fusion product 1	8	...	...	D7
BG078688	C	Dtnb	Dystrobrevin, beta	12	...	...	D7
BG086001	C	Sv2a	Synaptic vesicle glycoprotein 2 a	3	...	...	D7
BG073641	C	Rpl27	Ribosomal protein L27	11	D2	...	...
BG087402	C	Hnrpu	Heterogeneous nuclear ribonucleoprotein U	1	...	...	D7
595925	C	Sell	Selectin, lymphocyte	1	D2	...	...
1345776	C	Rbm14	RNA binding motif protein 14	19	...	...	D7
BG077327	C	Gins4	GINS complex subunit 4 (Sld5 homolog)	8	D2	D5	...
387319	C	Zfp346	Zinc finger protein 346	13	D2^e^	...	D7
AW546565	C	Exosc7	Exosome component 7	9	...	...	D7
BG072456	C	Pih1d1	PIH1 domain containing 1	7	...	...	D7
BG074521	C	Npm1	Nucleophosmin 1	11	D2	...	...
BG085278	C	Rpl22	Ribosomal protein L22	4	D2^e^	D5	D7
1445843	C	Ahcyl1	S-adenosylhomocysteine hydrolase-like 1	3	D2	...	D7
BG087592	C	Fbxw7	F-box and WD-40 domain protein 7, archipelago homolog (Drosophila)	3	D2	D5^e^	D7
BG064589	C	Atp6v1a1	ATPase, H+ transporting, lysosomal 70 kD, V1 subunit A, isoform 1	16	D2	...	D7
441227	C	Abtb1	Ankyrin repeat and BTB (POZ) domain containing 1	6	D2	D5	...
386911	C	Trim29	Tripartite motif protein 29	9	...	...	D7
BG087083	C	Serinc3	Serine incorporator 3	2	D2	...	...
372340	C	Myod1	Myogenic differentiation 1	7	D2	...	...
BG081915	C	Ube3a	Ubiquitin protein ligase E3A	7	D2	D5	...
573124	C	Mrrf	Mitochondrial ribosome recycling factor	2	D2	D5	...
573230	C	4930455C21Rik	RIKEN cDNA 4930455C21 gene	16	...	...	D7
1243507	C	Unknown	Unknown	...	D2	D5	D7
BG065706	C	Rabl3	RAB, member of RAS oncogene family-like 3	16	...	...	D7
BG078879	C	Cox4i1	Cytochrome c oxidase, subunit IVa	8	D2	...	...
BG088493	C	Ptplad1	Protein tyrosine phosphatase-like A domain containing 1	9	...	...	D7
1378435	C	Acad8	acyl-Coenzyme A dehydrogenase family, member 8	9	D2	...	...
1429286	C	1600002K03Rik	RIKEN cDNA 1600002K03 gene	10	...	D5	...
BG078872	C	Pfkfb2	6-phosphofructo-2-kinase/fructose-2,6-biphosphatase 2	1	...	D5	...
420477	C	Col13a1	Procollagen, type XIII, alpha 1	10	D2	...	...
402631	C	Crebl1	cAMP responsive element binding protein-like 1	17	...	D5	...
1445700	C	4930506M07Rik	RIKEN cDNA 4930506M07 gene	19	D2^e^	...	D7
575274	C	Nvl	Nuclear VCP-like	1	D2	...	D7
574369	C	Il4	Interleukin 4	11	D2	D5	D7
BG071511	C	Suclg1	Succinate-CoA ligase, GDP-forming, alpha subunit	6	D2	...	D7
573178	C	Igfbp7	Insulin-like growth factor binding protein 7	5	...	D5	...
574126	C	Dnaja3	DnaJ (Hsp40) homolog, subfamily A, member 3	16	D2	...	D7
1226596	C	Fem1c	fem-1 homolog c (C.elegans)	18	D2^e^	D5	D7
BG087153	C	Bckdha	Branched chain ketoacid dehydrogenase E1, alpha polypeptide	7	D2^e^	D5	D7
402019	C	Golga7	Golgi autoantigen, golgin subfamily a, 7	8	D2^e^	...	D7
574168	C	Prosc	Proline synthetase co-transcribed	8	D2^e^	D5	D7
BG073436	C	Atp5b	ATP synthase, H+ transporting mitochondrial F1 complex, beta subunit	10	D2^e^	D5	D7
574027	C	Med19	Mediator of RNA polymerase II transcription, subunit 19 homolog (yeast)	2	D2	D5	...
1243989	C	Mfhas1	Malignant fibrous histiocytoma amplified sequence 1	8	D2	D5	D7
373233	C	Itgb5	Integrin beta 5	16	...	...	D7
BG085816	C	Spna2	Alpha-spectrin 2, brain	2	...	...	D7
1446130	C	2410166I05Rik	RIKEN cDNA 2410166I05 gene	4	D2	...	D7
1263575	C	Stard4	StAR-related lipid transfer (START) domain containing 4	18	D2	D5	...
1852943	C	Ankrd17	Gene trap ankyrin repeat	5	...	...	D7
389112	C	Palm	Paralemmin	10	...	...	D7
619816	C	Smad5	MAD homolog 5 (Drosophila)	13	...	...	D7
641790	C	BC023814	cDNA sequence BC023814	3	...	...	D7
1329743	C	S3–12	Plasma membrane associated protein, S3–12	17	...	...	D7
653795	C	Il4ra	Interleukin 4 receptor, alpha	7	D2	...	D7
640435	C	Igsf3	Immunoglobulin superfamily, member 3	3	...	...	D7
387990	C	0610040D20Rik	RIKEN cDNA 0610040D20 gene	9	D2	D5	...
BG075962	C	Peci	Peroxisomal delta3, delta2-enoyl-Coenzyme A isomerase	13	D2	...	...
BG086412	C	Rpl31	Ribosomal protein L31	1	D2	...	...
BG075914	C	Rps9	Ribosomal protein S9	7	D2	...	...
639852	C	Bsg	Basigin	10	D2	...	...
573106	C	Slc25a1	Solute carrier family 25 (mitochondrial carrier; citrate transporter), member 1	16	D2	...	D7
574123	C	Suds3	Suppressor of defective silencing 3 homolog (S. cerevisiae)	5	D2	...	...
BG088819	C	1810043G02Rik	RIKEN cDNA 1810043G02 gene	10	D2	...	...
BG088858	C	1810044A24Rik	RIKEN cDNA 1810044A24 gene	15	D2	D5	D7
BG078275	C	C230096C10Rik	RIKEN cDNA C230096C10 gene	4	D2	...	D7
573120	C	2310035K24Rik	RIKEN cDNA 2310035K24 gene	2	D2	D5	...
BG076791	C	Eef1d	Eukaryotic translation elongation factor 1 delta (guanine nucleotide exchange protein)	15	D2	...	...
BG087946	C	Ubtf	Transcription factor UBF	11	D2	D5^f^	D7
372316	C	Ly6h	Lymphocyte antigen 6 complex, locus H	15	D2	...	D7
1243611	C	Tmem23	Transmembrane protein 23	19	D2	D5	D7
574299	C	2700059D21Rik	RIKEN cDNA 2700059D21 gene	4	...	D5	D7
BG064820	C	Sae1	SUMO1 activating enzyme subunit 1	7	D2	...	...
BG071827	C	Ppm1b	Protein phosphatase 1B, magnesium dependent, beta isoform	17	D2	D5^f^	...
BG086606	C	Txndc12	Thioredoxin domain containing 12 (endoplasmic reticulum)	4	D2	...	...
BG085962	C	Gsn	Gelsolin	2	D2	...	...
575308	C	Dus3l	Dihydrouridine synthase 3-like (S. cerevisiae)	17	...	...	D7
573228	C	Fyco1	FYVE and coiled-coil domain containing 1	9	D2	D5	...
BG081532	C	Sfpq	Splicing factor proline/glutamine rich (polypyrimidine tract binding protein associated)	4	D2	D5^f^	D7^f^
BG072614	C	Rps16	Ribosomal protein S16	7	D2	...	...
BG078928	C	Stmn3	Stathmin-like 3	2	...	...	D7
1226306	C	Cd274	CD274 antigen	19	D2	D5	...
AW544628	C	Itgb1	Integrin beta 1 (fibronectin receptor beta)	8	D2	...	D7
BG087322	C	Rps6ka2	Ribosomal protein S6 kinase, 90 kD, polypeptide 2	17	D2	D5	...
641964	C	Cd4	CD4 antigen	6	D2	D5^f^	...
BG071505	C	Slc28a3	Solute carrier family 28 (sodium-coupled nucleoside transporter), member 3	13	D2	D5	...
BG088775	C	Znhit3	Zinc finger, HIT type 3	11	D2	D5	...
BG069977	C	Pmm1	Phosphomannomutase 1	15	D2	D5	...
574022	E	1200015M12Rik	RIKEN cDNA 1200015M12 gene	3	D2	...	...
BG086286	E	Cirbp	Cold inducible RNA binding protein	10	D2	...	...
598493	E	Ifit3	Interferon-induced protein with tetratricopeptide repeats 3	19	D2	D5	...
617022	"	"	"	"	"	"	"
1329893	E	2600010E01Rik	RIKEN cDNA 2600010E01 gene	2	...	...	D7
BG076479	E	Ctla2a	Cytotoxic T lymphocyte-associated protein 2 alpha	13	...	D5^f^	D7
BG088327	E	Sf3b1	Splicing factor 3b, subunit 1, 155 kDa	1	...	D5	...
BG076355	E	Stat3	Signal transducer and activator of transcription 3	11	D2	...	...
643048	E	Gbp6	Guanylate binding protein 6	3	...	D5	...
638199	E	H2-T22	Histocompatibility 2, T region locus 22	17	D2	D5^f^	...
638232	E	Jundm2	Jun dimerization protein 2	12	D2	D5	...
1394984	E	Rps14	Ribosomal protein S14	18	...	...	D7

^a ^Clone ID or GenBank accession no.

^b ^The genes *Il4ra *and *Ifit3 *were duplicates (the same clone for the gene *Il4ra *and two different clones for the gene *Ifit3*). Thus, there were 177 genes identified.

^c ^Chromosomal localization.

^d ^The days for which the Welch t test was significant (<0.0001) after Bonferroni correction are mentioned.

^e ^Significant difference was observed only between BALB/c and C57BL/6.

^f ^Significant difference was observed only between BALB/c and CBA/J.

**Table 3:** Functional KEGG annotation of genes associated with resistance or susceptibility to CM

KEGG pathways^a^	Percentage of significant CM genes^b^
Ribosome	13.3
Cell adhesion molecules (CAMs)	10.0
Antigen processing and presentation	8.3
Oxidative phosphorylation	6.7
Hematopoietic cell lineage	6.7
Leukocyte transendothelial migration	6.7
ECM-receptor interaction	6.7
Cytokine-cytokine receptor interaction	5.0
Glycolysis/Gluconeogenesis	5.0
Fructose and mannose metabolism	5.0
Regulation of actin cytoskeleton	5.0
T cell receptor signaling pathway	5.0
Jak-STAT signalling pathway	5.0
Axon guidance	5.0
Valine, leucine and isoleucine degradation	5.0
Focal adhesion	5.0
Tight junction	5.0
MAPK signaling pathway	3.3
B cell receptor signaling pathway	3.3
Tryptophan metabolism	3.3
Propanoate metabolism	3.3
Butanoate metabolism	3.3
Bile acid biosynthesis	3.3
Fatty acid metabolism	3.3
Adipocytokine signaling pathway	3.3
Neuroactive ligand-receptor interaction	3.3
Neurodegenerative Disorders	3.3

^a ^Only the KEGG pathways that contained at least two genes were represented.

^b ^Of the 177 significant CM genes, 60 genes were annotated.

We further analyzed the expression of genes involved in neuronal development or in neurodegenerative disorders using immunochemistry. Thus, we studied the expression of *reelin *(*Reln*), which is involved in neurogenesis, and we searched for the presence of β-amyloid protein, which is involved in Alzheimer's disease. We detected RELN only in brains of BALB/c mice at days 5 and 7, while we showed the presence of β-amyloid only in brains of CBA/J and C57BL/6 mice at days 5 and 7 (Table 4).

**Table 4:** Protein expression in brain of CM-R and CM-S mice upon malaria infection

	d0/d2			D5			d7		
	BALB/c	CBA/J	C57BL/6	BALB/c	CBA/J	C57BL/6	BALB/c	CBA/J	C57BL/6

Reelin	-	-	-	++	-	-	+++	-	-
β-Amyloid	-	-	-	-	+	+	-	++	++

n = 3, at least 25 brain fields analysed per mouse.

-: no positive vessel

+: from 1 to 5 postive areas/vessels per field

++: from 6 to 10 postive areas/vessels per field

+++: > 10 positive areas/vessels per field

d0/d2: results were similar for both day 0 and day 2.

Overall, our gene expression analysis revealed marked changes in metabolic energy pathways, the inflammatory response, and pathways related to neurogenesis and neurodegenerative disorders in CM-S mice versus CM-R mice.

## Discussion

In the present study, we have searched for genes and physiological pathways potentially involved in CM. To this aim, we performed a longitudinal analysis of differentially expressed genes in brains from well-defined genetically CM-R (BALB/c) and CM-S (C57BL/6, and CBA/J) mice at early and late stages of infection. The present study shows that PbA strongly altered gene expression in these mice. In particular, gene expression was deeply altered at the time of CM onset, confirming our previous study [8]. Here, we show that a number of genes were over-expressed in CM-R mice at the early stage of infection, suggesting that CM-R mice mount an early protective transcriptional response. In this way, we found an association of resistance to CM with an increase in the expression of a number of genes on day 2 post-infection (Figure 4 and Table 2). In contrast, a number of genes were found to be under-expressed in C57BL/6 mice at the early stage of infection, while few genes were found to be regulated in CBA/J mice at the same time (Figure 2). Also, CBA/J mice appeared to mount a gradual response that may be involved in CM pathogenesis, while C57BL/6 mice may mount two waves of transcriptional responses both of them potentially implicated in malaria pathogenesis. These observations are consistent with other reports suggesting that CM mediators partly differ between C57BL/6 and CBA/J mice [8-10]. Nevertheless, both CBA/J and C57BL/6 mice showed a pronounced up-regulation of genes involved in either interferon-associated response or in glycolysis at the late stage, and a down-regulation of genes involved in erythropoiesis both at early and late stages, as previously described [3].

To identify genes with significant transcriptional changes associated with CM, we performed, on the whole data set, a multi-class SAM procedure with a very stringent false discovery rate followed by a Welch t test with a Bonferroni correction. In other words, we applied a two-filtering procedure to decrease the number of "false positive" genes. To account for natural variation between mouse strains, we adjusted gene expression level in infected mice for gene expression level in uninfected mice. Thus, we identified a number of genes differentially regulated between CM-R and CM-S mice. The 327 most differentially expressed genes identified by the SAM analysis allowed the complete discrimination between CM-R and CM-S mice according to the time of infection. The same result was obtained with the subset of 177 genes identified by the Welch t test (data not shown). This further confirms our previous study that investigated gene expression at the time of CM onset [8].

EASE analysis of either the 327 genes or the 177 genes revealed that some of the most represented biological process categories were related to the "defense response", such as the "response to parasite" or the "inflammatory response" terms. This was further supported by the results of the KEGG pathway analysis. In addition, genes were found to be involved in KEGG pathways related to metabolism, such as "oxidative phosphorylation", "glycolysis/gluconeogenesis", or "tryptophan metabolism". The analysis of functional annotation also revealed GO terms and KEGG pathways related to brain, such as the "axon guidance" and the "neurodegenerative disorders" KEGG pathways.

Overall, the analysis of functional annotation is consistent with the view that mouse CM is characterized among others by the deregulation of both immune response and glucose metabolism [11,12]. This leads to an abnormal increase in the inflammatory response and to hypoglycemia and acidosis in CM-S mice [11,12]. In addition, our data provide evidence of an intrinsic deficiency in oxidative phosphorylation, and the functional annotations related to brain disease suggest the role of genes expressed by brain cells in resistance or susceptibility to CM. Thus, despite a number of genes identified by our microarray analysis, which may highlight highly complex interactions between the parasite and the host, several major features of the transcriptional profile can be deduced.

First, PbA infection affects the expression of genes involved in metabolic energy pathways. The expression of *Uqcrq, Cox4i1, Ndufb8, Atp6v1g2 *and *Atp5b *involved in oxidative phosphorylation was upregulated in CM-R mice on day 2 post-infection [see Additional file 2], suggesting that cerebral oxidative metabolism may be stimulated by PbA infection in CM-R mice. In contrast, these genes were downregulated in C57BL/6 mice on day 2 post-infection and in CBA/J mice on day 7 post-infection, while only *Ndufb8 *was downregulated in CBA/J mice on day 2 post-infection. These transcriptional changes were associated with CM. A similar pattern was observed for *Hmgcs1 *involved in ketone metabolism. In the same way, lower NAD+/NADH levels and decreased mitochondrial function have been observed in CM-S mice by others [12,13]. These observations may be related to hypoxia and hypoglycemia, which reflect the low level of metabolic energy substrates. Alternatively, our data are consistent with the "cytopathic hypoxia" hypothesis, which rather proposes an adequate oxygen supply but an abnormal oxygen use [14]. Also, the low level of oxidative phosphorylation gene expression and ketone bodies pathway genes that we detected in the brain of CM-S mice suggests that cerebral oxidative metabolism may be inhibited even without oxygen delivery being impaired.

This metabolic disturbance also leads to lactate production and acidosis. In addition, this leads to an accumulation of ADP, which favors platelet aggregation [15]. Interestingly, platelet aggregation is known to be stimulated by PAF acether and inhibited by AMPc, the expression of which is inhibited by LIS1 (PAFAH1B1) and PDE4B, respectively [16,17]. Overall, the lower expression of *Pafah1b1 *in CM-S mice compared to CM-R mice [8], the higher expression of *Pde4b *in CM-S mice compared to CM-R mice, and the metabolic disturbance leading to an accumulation of ADP may participate in the platelet aggregation process in the cerebral microvasculature of CM-S mice.

Second, it is likely that the inflammatory response plays a major role in CM pathogenesis. In particular, a surge of IFNγ production at 3 to 4 days p.i. was demonstrated to be essential for murine CM, and this may be due to the absence of regulation in IFNγ pathways at early stages in PbA infection [18-20]. IFNγ is a proinflammatory cytokine typically produced by Th1 lymphocytes, and it is thought that the Th2 response protects from CM [11]. In this way, our microarray analysis showed that *IL4 *and *IL4R *were over-expressed in CM-R mice from day 2. Similarly, *Dnaja3, Foxo3a*, and *Ptpns1 *that inhibit the activity of NF-kB [21-23] had lower expression levels in CM-S mice than in CM-R mice, while *Nfkbia*, a marker of the NF-kB signalling pathway involved in inflammation [24], was over-expressed in CM-S mice. In addition, *C1qa *and *Pde4b *that are involved in the inflammatory response [17,25] were found to be over-expressed in CM-S mice compared to CM-R mice. *S100a10 *that inhibits the activity of phospholipase A2 [26], and *Bcl6 *that inhibits the production of MIP-alpha and IP-10 [27] were under-expressed in CM-S mice compared to CM-R mice. Blood cells are known to be involved in the inflammatory response due to malarial infection. Indeed, CD4+ and CD8+ T lymphocytes, platelets, monocytes have been shown to cooperate in the cerebral microvasculature, and this causes inflammation, endothelial cell damage, and hemorrhages [9,20,28-30]. In addition, *Gzmb*, whose expression was up-regulated in CM-S mice [8], encodes granzyme B in cytotoxic T lymphocytes, and is thought to be involved in the breakdown of the blood-brain barrier [31]. The influence of immune responses on the blood-brain barrier may be partly reflected by changes in expression of genes involved in either cytoskeletal and tight-junction pathways or cell adhesion pathways in CM-S mice.

It has been also suggested that glial cells actively participate in the local inflammatory response caused by malarial infection [32]. In this way, glial cells that have been shown to be activated on day 3 post-infection by PbA can produce C1q components [32,33]. The NF-kB signalling pathway has been demonstrated in these cells [34]. Interestingly, *Cd200 *that is implicated in the control of the activation of glial cells was strongly under-expressed in the CBA/J CM-S mice [35]. Besides, the expression of *IL4 *and *IL4R *was shown to be higher in CM-R mice than in CM-S mice. Since IL-4 induces apoptosis in activated glial cells that express IL-4R [36], IL-4 may contribute to the down-regulation of brain inflammation in CM-R mice.

**Figure 7:**
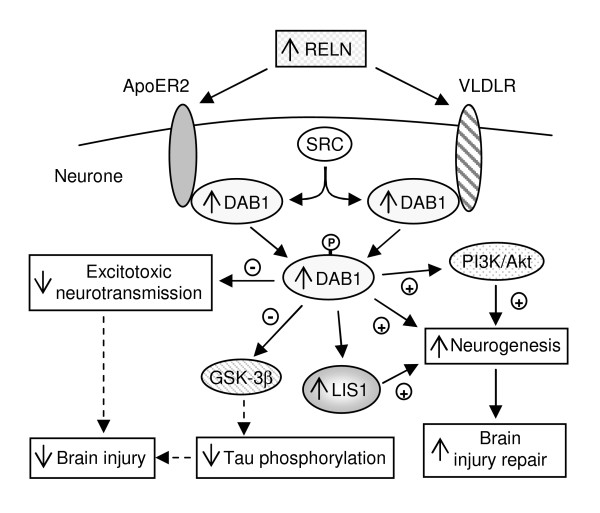
**Schematic diagram showing the possible effects of the reelin pathway in protection from CM**. Reelin (RELN) is an extracellular matrix serine protease expressed in some neurons, such as GABAergic interneurons, which inhibit excitotoxic neurotransmission [45]. RELN that is secreted into the extracellular space acts by paracrine and autocrine mechanisms. RELN interacts with very low-density lipoprotein receptors (VLDLR) and apolipoprotein E type 2 receptors (ApoER2) leading to tyrosine phosphorylation of the adaptor protein Disabled-1 (DAB1) by the SRC family kinases (SRC) [42]. DAB1 activation, in turn, activates PI3K/Akt signalling, which has been implicated in neuronal migration during development and adulthood. In addition, phosphorylated DAB1 interacts with LIS1, a protein encoded by *Pafah1b1*, which associates with microtubules and modulates neuronal migration [46]. LIS1 may be required for regulating crucial steps of reelin-dependent neuronal positioning. In parallel, phosphorylated DAB1 inhibits glycogen synthase kinase 3β (GSK3β), a kinase known to phosphorylate Tau protein at multiple sites. Therefore, the activation of RELN pathway diminishes the level of hyperphosphorylated Tau protein, which is a biomarker of brain injury. In particular, hyperphosphorylated Tau protein is a component of the neurofibrillary tangles involved in Alzheimer's disease. *Reln*, *Dab1 *and *Pafah1b1 *were shown to be over-expressed in CM-R mice compared to CM-S mice. The activation of RELN signalling may inhibit excitotoxic neurotransmission and Tau phosphorylation, and may activate neurogenesis in CM-R mice. This may lead to diminished brain injury and to increased brain injury repair. Solid arrows represent influences on the activity of proteins or physiological mechanisms. Dashed arrows represent impaired effects on the activity of proteins or physiological mechanisms. Negative signs indicate inhibition, and positive signs indicate activation.

Third, genes involved in the neuroprotection/neurotoxicity balance and/or in neurogenesis may protect the host against CM. This hypothesis is based on changes in tryptophan metabolism caused by PbA infection. Sanni et al (1998) showed an increase of the activity of indoleamine 2,3-dioxygenase whose expression is induced by TNF and IFNγ [37]. This leads to an increase of the ratio quinolinic acid/kynurenic acid, and to neuro-excitotoxic damage associated with CM. In humans, high levels of quinolinic acid have been associated with CM [38]. Interestingly, genes involved in tryptophan metabolism, such as *Ube3a, Prnt3*, and *Aldh1b1*, were differentially regulated between CM-R and CM-S mice. The expression of these genes was enhanced by infection in CM-R mice. In addition, genes having a neuroprotective role, such *Agtr2*, *Bag1*, *Csnk1a1 *and *Reln *[39-42], were shown to be over-expressed in CM-R mice compared to CM-S mice. Indeed, *Rtn3 *and *Creb1 *that are markers of neuronal survival [43,44], were also over-expressed in CM-R mice. Besides, *Reln *and *Dab1*, which were over-expressed in CM-R mice, are known to be involved in neurogenesis [45,46]. Similarly, *Pafah1b1 *whose expression was associated with resistance to CM [8] is involved in the *Reln *pathway [46]. This suggests that CM-S mice are deficient in neurogenesis, and that they cannot repair neuronal damages. In contrast, CM-R mice might be able to repair such damage (Figure 7).

So far, little research has been conducted on the issue of neuroprotective responses in CM. Interestingly, the *Reln *pathway that inhibits the phosphorylation of the protein Tau has been recently proposed as a protective mechanism against Alzheimer'disease [47]. In addition, decreased ribosomal RNA levels and decreased rates for protein synthesis have been recently described in Alzheimer'disease [48], while we showed the down-regulation of several genes encoding ribosomal proteins in CM-S mice. In the same way, Medana et al (2002) detected β-amyloid precursor protein in humans with CM [49], and we report here the β-amyloid protein in brains of CM-S mice but not in CM-R mice. This result is consistent with the down-regulation of *Arc, Itm2b, Bsg, Rtn3*, and *Il4 *in CM-S mice, and with the up-regulation of *Pde4b *in CM-S mice. Indeed, *Itm2b, Bsg, Rtn3*, and *Il4 *inhibit the production of the β-amyloid protein [50-53]. Besides, the β-amyloid protein inhibits the expression of *Arc*, while it increases the expression of *Pde4b *[25,54]. These observations suggest that cerebral malaria and Alzheimer'disease share some common mechanisms of pathogenesis.

## Conclusion

In this study, we have confirmed that gene-expression profiling discriminates between CM-R and CM-S mice, and we have identified genes whose expression showed consistent differential expression between CM-R and CM-S mice at early and late stages of infection. The analysis of gene functional annotation reveals several major features. First, it indicated that brain metabolic energy metabolism was early and deeply disturbed in CM-S mice, suggesting that high lactate production may be due rather to metabolic disturbance than to deficient oxygen supply. Second, the influence of inflammatory response on CM was also clearly detected, and our data are consistent with an active role of microglial cells in local inflammation. Third, the outcome of infection may critically depend on either cerebral tissue protective responses or brain repair capacity. Overall, our microarray analysis may give a global overview of critical events occurring in CM-S mice compared to CM-R mice. Searching for polymorphisms that alter the expression of genes identified should help in determining the genetic control of cerebral malaria. This analysis also revealed some promising areas for exploration that may both provide new insight into the key events that govern CM pathogenesis and the development of therapeutic strategies. In particular, novel neuroprotective therapies may be proposed as adjuncts to anti-malarial therapy.

## Methods

### Mouse strains and phenotyping

Six to 8 weeks old BALB/c (CM-R strain), C57BL/6J and CBA/J (CM-S strains) female, were obtained from IFFA CREDO (Ch. River Lab, France) and kept in our facilities. Three mice from each strain were not infected, and 22 BALB/c, 17 C57BL/6J, and 15 CBA/J were infected by i.p. injection of 10^6 ^*PbA *parasitized erythrocytes. The frozen stabilates were obtained with an uncloned line, and were prepared from CBA/J day 7-infected mice [30]. The parasite was conserved as stabilates of 10^7 ^parasitized erythrocytes stored in liquid nitrogen in Alsever's solution containing 10% glycerol. Parasitemia was monitored daily by blood smear. No difference was observed between mouse strains. Parasitemia was 0.8%±0.9, 4.5%±2.2, and 8.7%±5.3 on days 2, 5, and 7 post-infection, respectively. The CM-S mice developed a neurological syndrome (mono-, hemi-, para-, or tetraplegia, ataxia, deviation of the head, and convulsions), which occurred 6 to 7 days after parasite inoculation with a cumulative mortality of 100%. The CM-R mice did not present neurologic lesions and died during the 3^rd ^or the 4^th ^week of infection, with severe anaemia and hyperparasitemia [9]. The parasitemia of CM-R mice was 16.6%±8.9 and 62.8%±25.7 on days 9 and 15, respectively.

### Organ sampling

Brains were taken from CM-R and CM-S mice before and after infection. Brains from three uninfected mice were taken for each strain. Three, 4, and 6 CBA/J were analyzed on days 2, 5, and 7 post-infection, respectively, while 4, 4, and 9 C57BL/6 were analyzed on days 2, 5, and 7 post-infection, respectively. Brains from 3, 4, 5, 5, and 5 BALB/c mice were taken on days 2, 5, 7, 9 and 15 post-infection. Brains were completely removed and were cut into two parts: one part was frozen in RNALater (Qiagen, TM) until RNA analysis, and the other part was embedded in Tissue Tek (Leica), snap frozen in liquid nitrogen, and kept at -80°C until histopathological analysis of cryosections.

### Immunochemistry

For immunostaining, frozen sections were incubated for 45 minutes with primary monoclonal antibodies directed against murine Reelin and β-Amyloid peptide (Santa Cruz, Tebu Bio) after saturation with appropriate serum. After washing, sections were incubated for 45 minutes with biotinylated polyclonal antibodies, followed by the addition of HRPO-avidin (anti-rat or anti-hamster ABC kits; Vector, Peterborough, England). Color reaction was obtained by the addition of Novared (AbCys). Slides were counterstained with Mayer's hematoxylin before permanent mounting with Entellan (Merck, Brussels, Belgium). Slides were pictured at 200 magnification using an Eclipse 800 microscope (Nikon, Champignysur- Marne, France) and a digital camera; labelling was then analyzed by quantitative digitalized image analysis using Lucia software (Nikon). At least 3 brains were sampled for each time point and each mouse strain and image analysis was performed on an average of 25 microphotographs per mouse.

### RNA isolation and cDNA microarray hybridizations

Total RNA from brains was extracted using TRIzol reagent (Gibco-BRL, Life Technologies). The quality of RNA was confirmed on a formaldehyde agarose gel, and the concentration of RNA was determined by reading absorbance at 260/280 nm. RNA from 2 CBA/J mice had an inadequate quality, and was not further processed. Each mRNA sample extracted from an individual brain was run on a single microarray. In addition, three samples were run on several microarrays, and were considered as technical replicates: samples from CBA/J, C57BL/6, and BALB/c mice were run on 5, 2, and 2 microarrays, respectively. All microarray procedures were done at our microarray core facility [55]. cDNA microarrays were designed and prepared as described [56]. Briefly, the microarrays used in this study were composed of 8388 sequences. The following cDNA libraries were used: the NIA Mouse 15 K cDNA clone set, 2NbMT (thymus), NbMLN (lymph node), and 3NbMS (spleen). Detailed descriptions of these cDNA libraries are available at the UniGene database website (2NbMT: Lib.544, 3NbMS: Lib.553, NbMLN: Lib.567, NIA 15 K: Lib.8622) [57]. PCR amplification was performed as previously described [56], and PCR products were spotted onto nylon membranes (Hybond-N+, Amersham) with a MicroGrid II arrayer (Affymetrix, Santa Clara, CA). About 10% of the genes included in this clone set are represented by two or more different cDNA clones, providing internal controls to assess the reproducibility of gene expression measurements. Microarrays were hybridized with ^33^P-labelled probes, first with an oligonucleotide sequence common to all spotted PCR products (5'-TCACACAGGAAACAGCTATGAC-3'), then after stripping, with complex probes made from 5 μg of retrotranscribed total RNA. Probe preparations, hybridizations and washes were carried out as described previously [56]. After 48 h hybridization, arrays were scanned with a FUJI BAS5000 machine at 25 μm resolution. Hybridization signals were quantified using ArrayGauge software (Fuji Ltd, Tokyo, Japan).

### Microarray data analysis

All images were carefully inspected, and spots with overestimated intensities due to neighborhood effects were manually excluded. The data were filtered such that only spots with intensities that were two times greater than the median background in either microarray were used in the analysis, and the signal intensities were then corrected to take into account the amount of spotted DNA and the variability of experimental conditions, as described [58]. Of the 8388 spotted clones, we selected the clones that had detectable expression levels in at least 80% of the experiments (n = 2012). Unsupervised hierarchical clustering investigated relationships between samples and relationships between genes. It was applied to data log-transformed and median-centred using the Cluster and TreeView programs (average linkage clustering using Pearson's correlation as similarity metric) [59].

Microarray data were statistically analyzed using the TIGR MeV (MultiExperiment Viewer) v3.1 software [60]. Figure 1 shows an outline of data analysis. A one-way ANOVA and SAM (Significant Analysis of Microarrays) procedures were applied to look for time-, strain-, and CM-R/CM-S-specific variation in gene expression in the full data set. One-way ANOVA and Welch t-statistics were used to analyze gene expression changes upon infection for each mouse strain. The values on days 2, 5, 7, 9 and 15 post-infection were compared to values on day 0 before infection. To search for gene expression changes associated with CM, a multi-class SAM procedure and a Welch t test were performed on the log2 ratios of infected vs uninfected samples. For each gene, the level of gene expression in each sample taken from an infected mouse was divided by the median of gene expression levels in samples taken from three uninfected mice. This calculation was done for each mouse strain. Multiple test corrections were performed [61].

The Expression Analysis Systematic Explorer (EASE) was used to search for common biological themes within gene lists generated by our microarray analysis [62]. EASE assigns identified genes to Gene Ontology (GO) terms, and tests whether specific biological pathways were over-represented within specific gene clusters. A score based on Fisher Exact test reports the probability that the prevalence of a particular theme within a cluster is due to chance alone given the prevalence of that theme in the population of all genes under study. In addition, we checked whether the genes were included in a KEGG pathway [63].

All data are MIAME compliant and have been loaded into ArrayExpress database [64]. The ArrayExpress accession number of this experiment is E-MEXP-1018.

## Abbreviations

CM: Cerebral Malaria

CM-S: Cerebral Malaria-Susceptible mice

CM-R: Cerebral Malaria-Resistant mice

PbA: *Plasmodium berghei *ANKA

GO: Gene Ontology

SAM: Significant Analysis of Microarrays

RELN: Reelin

## Competing interests

The author(s) declare that they have no competing interests.

## Authors' contributions

NFD participated in the design of the study, the injection of parasitized erythrocytes, the sample preparation, and the analysis of the data, carried out microarray experiments, and prepared the figures. NC participated in sample preparation and carried out imunochemistry experiments. DP, FJ, and CN designed and produced the microarrays. DP participated in the statistical analysis, and CN contributed to study design. MB participated in microarray hybridizations. PB and GG participated in the interpretation of data that concerned genes involved in the inflammatory response. NFD and PR carried out a systematic analysis of the functional annotation of genes identified. FAI and GG participated in the design of animal studies. PR conceived and coordinated the study, and wrote the manuscript. All authors read and approved the final manuscript.

## Supplementary Material

Additional File 1 The full list of the 327 genes that discriminated between early and late infection stages, between mouse strains, and between CM-R and CM-S mice. Click here for file

Additional File 2 The graphical representation of expression profiles of genes involved in oxidative phosphorylation in CM-S and CM-R mice. Click here for file
