# Supplementary material for: Gene expression analysis reveals early changes in several molecular pathways in cerebral malaria-susceptible mice versus cerebral malaria-resistant mice
Source: BMC Genomics. 2007 Dec 6;8:452. doi: 10.1186/1471-2164-8-452 (PMC2246131; doi:10.1186/1471-2164-8-452)
Supplement: Additional File 2 — The graphical representation of expression profiles of genes involved in oxidative phosphorylation in CM-S and CM-R mice. [file 1471-2164-8-452-S2.pdf]

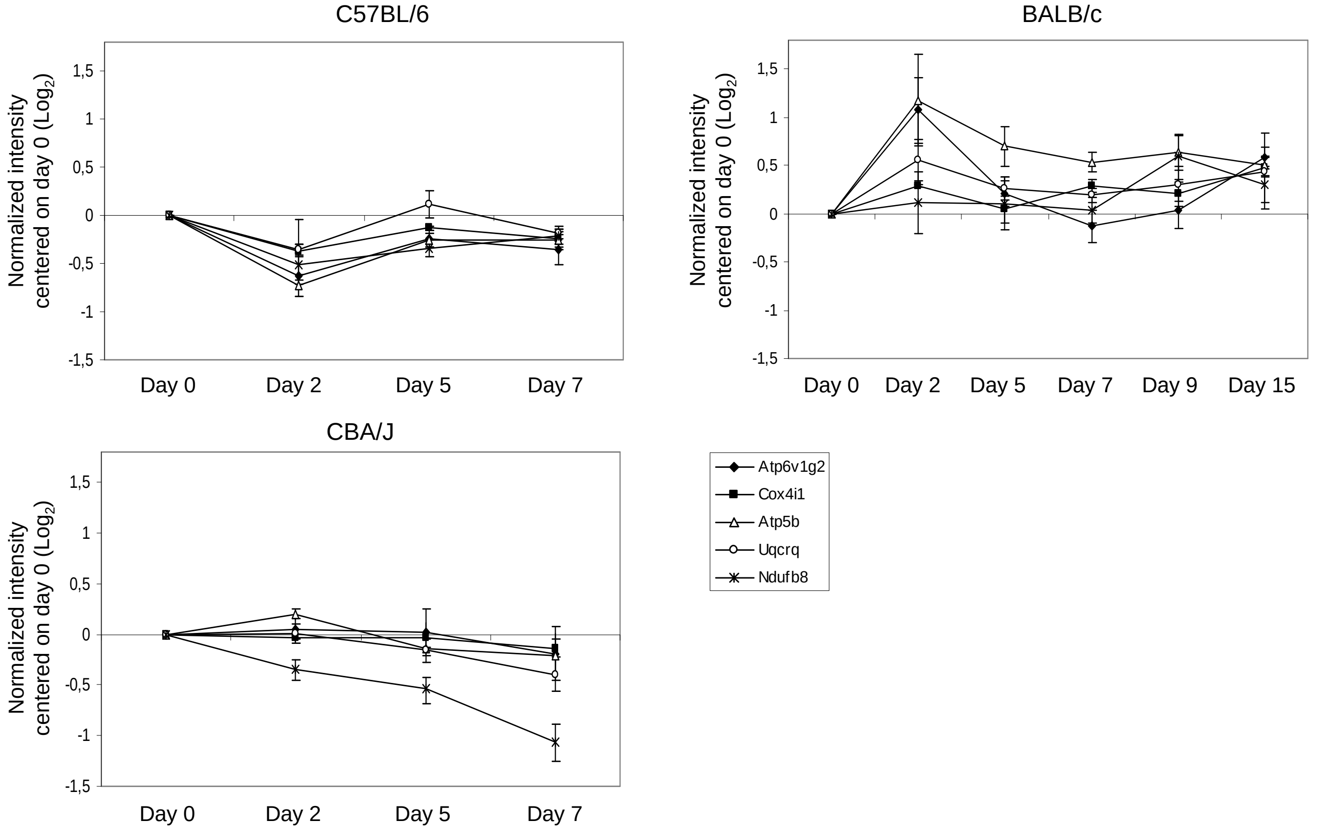

Additional file 2. Expression of genes involved in oxidative phosphorylation in CM-S and CM-R mice

Graphical representation of gene expression profiles based on the log2 ratios of infected vs uninfected mice (normalized intensities centered on day 0) in CM-S (C57BL/6 and CBA/J) and CM-R (BALB/c) mice upon PbA infection. Data for each time point represent the median values ± SE of mice groups. For C57BL/6 mice, n=3, 4, 4 and 10, on days 0, 2, 5 and 7, respectively. For CBA/J mice, n=3, 7, 4 and 6, on days 0, 2, 5 and 7, respectively. For BALB/c mice, n=3, 3, 4, 6, 5 and 5, on days 0, 2, 5, 7, 9 and 15, respectively.
